# Regulation and Evolution of NLR Genes: A Close Interconnection for Plant Immunity

**DOI:** 10.3390/ijms19061662

**Published:** 2018-06-05

**Authors:** Grazia M. Borrelli, Elisabetta Mazzucotelli, Daniela Marone, Cristina Crosatti, Vania Michelotti, Giampiero Valè, Anna M. Mastrangelo

**Affiliations:** 1 Council for Agricultural Research and Economics—Research Centre for Cereal and Industrial Crops, s.s. 673, km 25.2, 71122 Foggia, Italy; graziamaria.borrelli@crea.gov.it (G.M.B.); daniela.marone@crea.gov.it (D.M.); 2 Council for Agricultural Research and Economics—Research Centre for Genomics and Bioinformatics, via San Protaso 302, 29017 Fiorenzuola d’Arda (PC), Italy; elisabetta.mazzucotelli@crea.gov.it (E.M.); cristina.crosatti@crea.gov.it (C.C.); vania.michelotti@crea.gov.it (V.M.); 3 Council for Agricultural Research and Economics—Research Centre for Cereal and Industrial Crops, s.s. 11 to Torino, km 2.5, 13100 Vercelli, Italy; giampiero.vale@crea.gov.it; 4 Council for Agricultural Research and Economics—Research Centre for Cereal and Industrial Crops, via Stezzano 24, 24126 Bergamo, Italy

**Keywords:** *NLR* genes, gene regulation, gene evolution, plant breeding

## Abstract

NLR (NOD-like receptor) genes belong to one of the largest gene families in plants. Their role in plants’ resistance to pathogens has been clearly described for many members of this gene family, and dysregulation or overexpression of some of these genes has been shown to induce an autoimmunity state that strongly affects plant growth and yield. For this reason, these genes have to be tightly regulated in their expression and activity, and several regulatory mechanisms are described here that tune their gene expression and protein levels. This gene family is subjected to rapid evolution, and to maintain diversity at NLRs, a plethora of genetic mechanisms have been identified as sources of variation. Interestingly, regulation of gene expression and evolution of this gene family are two strictly interconnected aspects. Indeed, some examples have been reported in which mechanisms of gene expression regulation have roles in promotion of the evolution of this gene family. Moreover, co-evolution of the *NLR* gene family and other gene families devoted to their control has been recently demonstrated, as in the case of miRNAs.

## 1. Introduction

Both animals and plants cope with pathogen attacks, which are among the most important biotic stresses. While mammals have specialized cells to confer adaptive immunity, plants have large numbers of immune receptors in all cells. These are organized into different families, for sensing several pathogens [1]. Two main types of immune responses can be activated, which are based on the actions of receptors located both at the cell surface and inside the cell. The first type, as the basal response, acts through pattern recognition receptors (PRRs), which are plasma membrane localized and can recognize the so-called elicitors—the generic signals of the presence of a pathogen, such as bacterial flagellins or lipopolysaccharides, and fungal chitin or heptaglucosides [2]. These are referred to as microbe-associated molecular patterns (MAMPs), because they are common to pathogens and nonpathogenic microorganisms [3]. The second mechanism, known as effector-triggered immunity, is based on the specific recognition of pathogenic effectors through the action of receptors coded by resistance (*R*) genes. These receptors are mainly intracellular, and they can specifically interact with pathogen effectors coded by the avirulence (*Avr*) genes following the “gene for gene” model [4]. More recently, to explain resistance mechanisms that are not completely explained by direct interactions between a receptor and a pathogen effector, the guard and decoy hypotheses were proposed. In this model, the interaction between receptor and effector can occur directly through so-called decoy domains fused to NOD-like receptors (NLRs), or indirectly through an accessory plant protein, which can be the virulence target (“guard model”) or a structural mimic of such a target (“decoy model”) [5].

More than 100 *R* genes have been cloned from various species, and most of them contain a conserved nucleotide-binding domain (NB-ARC) and leucine-rich repeat domain (LRR) [6]. Plant NLR-mediated immune responses often culminate in a hypersensitive response, which is a programmed cell death event that leads to restricted biotrophic pathogen growth at the site of infection [1]. Typical plant NLRs can be further classified into two groups based on the presence at the N-terminus of the Toll-like/interleukin 1 domain (TIR-type NLRs) or a different domain (non-TIR-type NLRs), such as the coiled-coil domain non-TIR-type. These two NLR gene groups differ in their structural features, and also in their mode of action. In general, TIR-type NLRs require EDS1 (enhanced disease susceptibility 1) to activate the downstream signaling pathway, while non-TIR-type NLRs require NDR1 (non-race-specific disease resistance 1) [7]. Nonetheless, some exceptions have been reported, such as the non-TIR-type NLR Arabidopsis turnip crinkle virus (*HRT*) gene for resistance to TCV (turnip crinkle virus), which depends on EDS1 for signaling [8], while a few other non-TIR-type NLR genes are independent of both EDS1 and NDR1 [7,9,10].

As well as its importance for plant physiology and production, resistance to pathogens has a metabolic cost. Several studies have underlined that diversification of NLRs can have pleiotropic effects on plant fitness [11,12], while several NLR proteins involved in resistance to a particular pathogen can induce a reduction in plant fitness when the pathogen is absent. This is proposed to be a result of the metabolic cost of protein synthesis and regulation, or aberrant defense activation [13,14]. The activation of NLRs is thought to be regulated by the NB-ARC domain, which acts as a molecular switch. In more detail, when no pathogens are present, an NLR protein is in a resting state in which it is bound to ADP, and this state is stabilized by the LRR domain. When an effector is present, a conformational change occurs in the LRR domain, and consequently, in the NB-ARC domain, which releases the ADP. The subsequent binding of ATP activates the NLR, which triggers a defense reaction [15] (Takken et al., 2006). More recently, Bernoux et al. (2016) [16] proposed that NLRs exist in an equilibrium between on and off states. When an effector is present, it binds to the ON state and stabilizes its conformation. As a consequence, the equilibrium is shifted toward the active form of the receptor, to trigger defense signaling.

Moreover, tight regulation of NLR expression is necessary to avoid autoimmunity or retarded plant growth, while at the same time maintaining a prompt response to biotic stresses [17,18]. In *Arabidopsis thaliana*, two NLR genes, *DM1* and *DM2* (*DANGEROUS MIX 1* and *2*), genetically interact with each other and cause autoimmunity when hybrids are produced by crossing plants harboring specific alleles of the two NLRs [19].

The abundance and sequence variability of NLR genes are necessary features for the recognition of the many variable pathogens effectors. Several mechanisms have been shown to be involved in their evolution to maintain the recognition of different pathogen-derived avirulence (Avr) proteins. LRR domains, which are devoted to protein–protein interactions, can evolve very different binding specificities because of their highly adaptable structural features. Indeed, many studies have shown that LRRs are under diversifying selection [20,21], and in particular, the conservation at the level of the predicted solvent-exposed residues is lower than expected from random genetic drift. Other mechanisms contribute to the recognition of different pathogen effectors (discussed below), such as gene conversion and duplication events followed by local rearrangements. As the NLR gene family is subject to continuous evolution, the mechanisms of their regulation of expression also have to evolve in a coordinated manner. This aspect is crucial for plants to cope with a changing environment in which new and virulent strains of pathogens occur. These aspects are reviewed here, from gene family evolution to the fine regulation of *NLR* gene expression and protein activity, which takes place at different levels, from RNA synthesis/degradation to protein modification. The tight link between these regulation mechanisms and the evolution of the gene family is discussed.

## 2. Gene Family Organization

Comparative analyses of full-genome sequences of different plant species belonging to diverse phylogenetic groups have revealed that plant genomes harbor hundreds of NLR genes. The number of these genes is highly variable across species and within the same family or the same genus. In the next paragraphs, some biological explanations are provided for the observed differences. Nonetheless, it is important to highlight that these differences can be at least partly a result of the different approaches used for NLR identification. These approaches have been defined more precisely in recent years, as knowledge of the genome sequences has greatly increased and improved for several species. New bioinformatic pipelines to identify gene families in sequenced genomes have also been developed. A small number of NLR genes (50–100) has been defined for the *Carica papaya* genome [22], cucumber [23], and watermelon [24], compared with other species such as rice and grape, which have over 500 genes [25]. More than 2000 NLR-encoding genes have been identified in bread wheat, the largest number reported so far [26].

**Figure 1:**
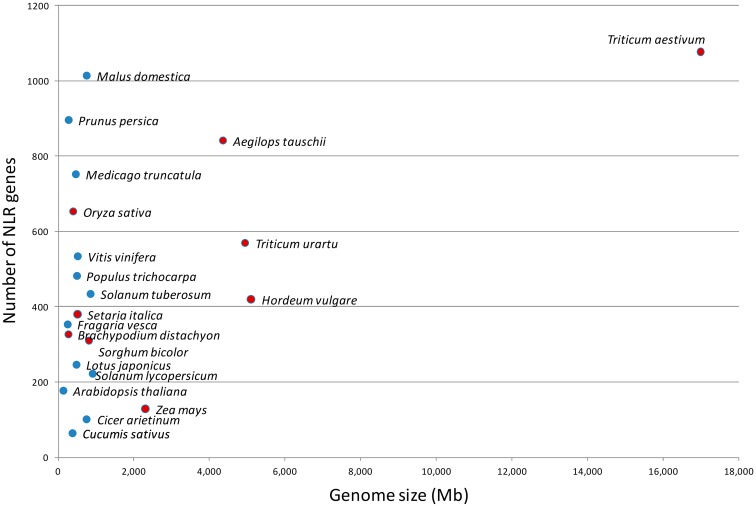
Number of NOD-like receptor (NLR) genes in relation to genome size in some monocot (red) and dicot (blue) species.

A first possible explanation of this variability is that the number of NLR genes is proportional to the size of the genome, but this does not appear to be the case (Figure 1), as nearly 1000 NLR genes have been identified in apple, even if its genome size is approximately 740 Mb [27]. The expansion of the NLR gene family in apple and other woody plants that are characterized by very long life, and probably greater exposure to pathogens, might compensate for a limited capacity to generate novelty due to infrequent meiosis [28,29,30].

Ploidy levels can also account for NLR expansion; in many lineages, the increase in NLR copy numbers is associated with polyploidy or ancient polyploidization events (e.g., in hexaploid wheat (*Triticum aestivum*) and in the recently duplicated apple (*Malus domestica*) genomes) [29]. Nevertheless, Levy and Feldman [31] reported that the polyploids of the *Gossypium* and *Oryza* genera have similar numbers of nucleotide binding site (NBS) genes to those of their donors, and concluded that polyploidization might lead to an immediate expansion of the NBS families, with the subsequent pseudogenization of many NLR genes. In general, it can be hypothesized that in plant species that have more NLRs, these can provide broader pathogen recognition, as well as a substrate of related sequences that allows for more frequent recombination [30,32].

Furthermore, there is also variation in the gene copy numbers of the two subclasses, TIR-type NLRs and non-TIR-type NLRs. To date, the former subgroup has not been described for the grass genome, whereas the latter is present in both monocots and dicots. Although all dicots have both non-TIR-type NLRs and TIR-type NLRs, some of them have unbalanced numbers of genes per class. For example, the non-TIR-type NLR to TIR-type NLR ratio is 1:2 in Brassicaceae and 4:1 in potato and grapevine [33], whereas a ratio of 1:1 has been reported for apple [34]. In both *Arabidopsis thaliana* and *A. lyrata*, all chromosomes harbor more TIR-type than non-TIR-type NLR genes [35].

The distribution of NLR genes is irregular across and along chromosomes, as they are more represented in some chromosomes than in others. Various mechanisms are believed to contribute to the diversity of NLR loci, including recombination, gene conversion, duplication, and selection. It is thus possible that different recombination frequencies along chromosomes affect their distribution [36]. Indeed, in several species, NLR genes are often located in subtelomeric regions, which are characterized by higher recombination frequency, as in common bean, in which three ‘super’ clusters have been located on the distal ends of chromosomes 4, 10, and 11 [37]. This peculiar location has also been observed in potato, tomato, cotton, and *Setaria italica* [38,39,40,41]. However, this peculiar location is not observed in all plant species, as it has not been reported in Arabidopsis or rice [37].

Moreover, in some cases, events such as chromosome translocations can also explain the uneven distribution of NLR loci across chromosomes. In *Brachipodium distachyon*, about one-third of the total NLR genes are located on chromosome 4 [42], whereas chromosomes 2 and 3 in *Lotus japonicus* contain higher numbers of these genes [43]. Kang et al. [44] reported the highest number of NLR genes on chromosome 16 of the soybean genome, while 96 genes containing TIR domains identified in the Chinese cabbage genome were mapped to nine of the ten chromosomes, with chromosome 9 as the most representative one [45]. Similarly, a non-random distribution of NLR genes among chromosomes has been shown for *Brassica rapa*, *Capsella rubella*, and *Thellungiella salsuginea* [35].

Within genomes, NLR genes are organized either as isolated genes, or as clusters of varying sizes, which are known as homogeneous when they contain sequences that share a common ancestor, and heterogeneous if they contain more distantly related NLRs [36]. Tandem duplications, in which the paralogs are contiguous to the original gene, are usually associated with homogeneous clusters. In contrast, segmental and ectopic duplications, which involve the duplication of entire gene blocks or single/small groups of genes, respectively, can explain the heterogeneous clusters. A cluster is defined when the distance between neighboring NLRs is less than 200 kb [38]. NLR gene clustering is a common phenomenon in plant genomes, even if the percentage of NLR genes organized into clusters is highly variable across plant species, from 27.3% in Brachypodium [6] to 90% in *Sorghum bicolor* [46]. The numbers and sizes of clusters are also different across plant species. One of the highest numbers of clusters has been identified for *M. domestica* (153), in which 80.7% of NLRs are thus arranged [34]. In some cases, as in chickpea, homogeneous clusters (78% of NLRs) are more abundant than mixed clusters [47], while in sorghum, the homogeneous clusters are smaller than the heterogeneous ones [6]. For the cluster sizes, an average of 33 genes per cluster was identified in *Eucaliptus grandis*, but this feature is also very variable across species [48].

The occurrence of NBS-LRRs in clusters indicates that gene duplication has had great impact on expansion and evolution of this gene family in plant genomes, providing a reservoir of genetic variation of NLR genes.

## 3. NLR Transcriptional Regulation

The first step in the regulation of NLR expression occurs at the transcriptional level. In several studies, the steady-state levels of NLR transcripts have been quantified through array-based and next generation sequencing of steady state RNA (RNA-seq) transcriptome analyses. Transcript levels for NLRs were shown to increase in response to different defence-associated treatments, such as MAMPs [49,50], and in response to pathogen infections [51]. The spatial-temporal regulation of NLR expression was addressed by a recent meta-analysis of NLR expression in different species in relation to the organs or tissues and growth stages in which the probability of encountering a pathogen is higher for a given species. As an example, the NLR expression level is higher in shoots compared with roots in Arabidopsis, while an opposite trend is observed in the legume Lotus. This can be explained by the ability of the latter to establish endomycorrhization interactions [52]. Even the time of day appears to influence the expression of NLR genes in relation to the circadian rhythm [53,54]. Other studies have underlined relationships between NLR expression levels and resistant phenotypes to a given pathogen. As an example, real-time quantitative polymerase chain reaction analysis indicated that NLR genes are expressed more in roots of coconut genotypes resistant to root (wilt) disease caused by phytoplasma than in susceptible ones [55]. Similarly, when the expression of several chickpea NLR genes was assessed in two resistant genotypes and one susceptible genotype at different times after Ascochyta blight infection, 27 NLR genes showed differential expression in at least one genotype at one time, and among these, five showed expression patterns that were specific for the genotype. Moreover, expression profiling of 30 NLR genes differentiated the three genotypes, as the susceptible cultivar was separated from the moderately resistant cultivars, owing to the overexpression and the time of up-regulation of some NLRs in the resistant and moderately resistant cultivars [48].

Transcription factors have important roles in transcriptional regulation of NLR genes by specifically recognizing motifs in their promoters. In the model species Arabidopsis and rice, upstream sequences of many NLR genes were analyzed to predict and characterize potential transcription factor binding motifs [56]. Several motifs were shown to be over represented in NLR promoters, which were known to be linked to transcriptional responses to biotic stresses. Other motifs were defined that were responsive to wounding, heat shock elements, water stress, and abscisic acid responses. Although most of these elements were conserved in the two species, species-specific motifs were also identified. Information about these motifs and their frequency and positional conservation might help to find co-expressed genes and to predict gene expression patterns based on the motif arrangement.

It is important to underline that the changes in steady-state transcript abundance observed in transcriptomic studies might be a result of changes in the rate of transcription and/or of transcript degradation. Overexpression of NLR genes coupled to post-transcriptional and post-translational regulation steps might allow plants to activate receptors very rapidly, to avoid the longer times required for de novo transcription of the genes.

Interestingly, interactions between NLR proteins and transcription factors can regulate downstream responses to pathogens. In barley, alleles of the intracellular immune receptor mildew A (MLA) confer resistance specificities against different *Blumeria graminis* strains. HvWRKY1 forms a nuclear complex with HvMYB6 that suppresses defense gene activation in the absence of the pathogen. After infection, the fungal-derived effector is recognized in the cytosol by the non-TIR-type domain of MLA, which then migrates to the nucleus and interacts with HvWRKY1, which releases HvMYB6, in order to allow its contact with DNA and to activate gene expression [57]. A recent study suggested that there might also be a role for MLA in resistance in the cytoplasm [58].

## 4. Regulation by Alternative Splicing and Nonsense-Mediated RNA Decay

Alternative splicing consists of the production of alternative transcript forms from the same gene following different patterns of splicing. This is a well-known post-transcriptional process that finely regulates the expression of genes both quantitatively and qualitatively in several biological processes, including responses to biotic and abiotic stresses [59]. In TIR-type NLR genes, the first exon encodes the TIR domain, and the second exon the NB domain, while the LRR region and the additional C-terminal parts are encoded by the remaining exons [60]. In some cases, as for the N or RPS4 TIR-type NLR genes, alternative splicing can produce transcripts that retain one intron with the introduction of premature in-frame stop codons in the mature mRNAs. The resulting proteins are thus truncated and partially or fully lack their LRR or other C-terminal portions. This can have effects on intramolecular (between individual NLR protein domains) or intermolecular (with signaling components) interactions, to regulate the NLR protein activity [61,62]. Indeed, in many cases, a functional role of these truncated polypeptides has been demonstrated for resistance to pathogens, as summarized in Mastrangelo et al. [59].

More recently, with the advent of genome-wide studies, pathogen-responsive alternative splicing events have been highlighted. Using RNA sequencing approaches, Mandadi et al. [63] generated an isoform-level spliceosome map of *B. distachyon* infected with Panicum mosaic virus and its satellite virus, and observed virus-induced alternative splicing events for more than 100 genes related to immune responses, among which there were nine NLR genes. Moreover, Zhang et al. [64] applied RNA-seq to inflorescences of the Arabidopsis wild-type and the *sr45–1* viable mutant. Arabidopsis Serine/Arginine-rich 45 (SR45) regulates pre-mRNA splicing by interacting with other regulatory proteins and spliceosomal subunits. Using bioinformatics tools, they identified 542 SR45-dependent alternative splicing events, and several out of these coded for NLR proteins. Indeed, *sr45–1* plants showed enhanced immunity, which might be because of changes in their NLR splicing patterns.

The majority of alternative splicing events related to pathogen attacks have been observed in TIR-type NLRs, probably because of the common structure shared by these genes in terms of their exon/intron organization. Despite this, some infection-related alternative splicing has also been reported for non-TIR-type NLRs, including *Sr35* in wheat [65], *Mla* in barley [66,67], *Pi-ta* and *RGA5* in rice [62,68], and *JA1tr* in common bean [69]. However, the functional meaning of the alternative splicing events in resistance has not always been clarified [60]. The rice *RGA5* gene is a well characterized system [68], in which two transcript isoforms are generated by alternative splicing of the third of three introns [70]; the isoforms differ only in their *C*-terminal regions, which contain the RATX1 domain (related to the *Saccharomyces cerevisiae* copper binding protein, ATX1) required for recognition of the *Magnaporthe oryzae* effector AVR1-CO39. The intron-retaining form was described as related to *M. oryzae* full susceptibility [68]. The wheat leaf rust resistance gene *Lr10* sequence, a non-TIR-type NLR gene [71], was analyzed in a collection of 58 wild emmer accessions from Israel, and two haplotypes with different splicing regulation motifs were identified as producing forms with retained introns. These two haplotypes are also present in durum wheat, and both confer resistance to leaf rust [72] and might be maintained by balancing selection [73]. Discovering genetic variation for alternative splicing events in resistance genes opens the possibility of selecting new sources of resistance based on alternative splicing.

The production of active truncated proteins is a sort of qualitative mechanism of regulation of the activity of the NLR proteins, but recent studies have also unraveled quantitative control of the final levels of functional proteins at the transcriptional level, through nonsense-mediated mRNA decay (NMD) [74]. This is a conserved eukaryotic RNA surveillance mechanism that degrades aberrant mRNAs. In recent years, NMD has emerged as a mechanism to finely regulate the levels of functional proteins in the cell in different biological processes, including responses to biotic stresses [75]. The inhibition of NMD elicits constitutive activation of plant immune responses [74]. Indeed, impaired function of the NMD proteins SMG7, UPF1, or UPF3 results in an autoimmune response, with activation of the plant defense mechanisms. This includes the expression of genes related to plant defense, the production of salicylic acid, and enhanced resistance to bacterial infection. Moreover, plants engineered to overexpress SMG7 fail to down-regulate NMD upon infection, and show increased sensitivity to pathogenic bacteria [74,76]. The autoimmunity is achieved through dysregulation of TIR-type NLR genes [76,77], and nearly one-third of these appear to be targeted by NMD in Arabidopsis [75]. A recent example [77] illustrates that the autoimmunity related to NMD deficiency might be dependent on different TNLs in different Arabidopsis accessions. In particular, regulation of the TNL RPS6 can trigger autoimmunity that results from NMD impairment in the *Landsberg erecta* ecotype, while different TNL genes might be involved in the same process in the Columbia ecotype.

The relationships between alternative splicing and NMD remain an open topic, as intron retention, which introduces premature stop codons to interrupt the open reading frame, and NMD might be coupled to regulate the levels of functional NLR proteins in response to biotic stresses, as already observed for proteins involved in responses to abiotic stresses [59]. Some studies have shown that plant transcripts with retained introns are often not subjected to NMD, as they are retained in the nucleus or nucleolus [78,79]. Moreover, in Arabidopsis, NMD impairment promotes the accumulation of a subset of RPS6 splice isoforms that are generated by the excision of a cryptic intron, while transcript variants that contain premature termination codons resulting from an intron retention event are not affected [77]. Moreover, premature termination codons generated by intron retention events generally do not elicit NMD in plants, because of nuclear retention of these transcripts [79]. A different indication is given by the AtGRP7 (*Arabidopsis thaliana* GLYCINE RICH RNA-BINDING PROTEIN 7) protein, which negatively auto-regulates by alternative splicing, to generate an isoform that contains a premature termination codon that decays via NMD [80,81]. AtGRP7 is part of the circadian timing system and has a role in pattern-triggered immunity and resistance against *Pseudomonas syringae* pv *tomato* DC3000 [82,83]. Further experimental evidence is needed to clarify the exact relationships between alternative splicing and NMD in responses to biotic stresses.

## 5. Regulation by Small RNAs

NLR-encoding genes are one of the largest families targeted by small RNAs. Among the small RNAs, the 22-nt-length miRNAs and the secondary structures of the miRNA duplex can trigger the generation of secondary small-interfering RNAs (siRNAs) from their target mRNAs [84]. Both miRNAs and secondary siRNAs act in the regulation of NLR gene expression to silence these immune receptors in the absence of disease and to restore their basal levels in the late phases of infection [85]. Recent evidence has indicated that plants have evolved specific miRNAs to target conserved domains in NLR-encoding genes, and that as well as miRNAs and secondary siRNAs, plants trigger the production of a phased array (in a sequential, head-to-tail manner, according to the miRNA cleavage site) of 21-nt secondary small-interfering RNAs (phasiRNAs) to amplify silencing effects. PhasiRNA-based regulation of the NLR genes is suggested to have roles in the stabilizing of their basal expression levels, to reduce the fitness costs of an overactive immune response that can have deleterious consequences for plant growth [84]. In this respect, the regulatory activity of small RNAs toward NLR genes is modulated during plant growth. In particular, high-throughput sequencing of small RNAs and mRNAs from different plant growth stages has revealed that most NLR genes in tomato and tobacco are regulated by small RNAs, with decreased expression levels of the small RNAs’ silencing system and corresponding increased expression levels of their NLR transcript targets in mature plants [86]. This behavior might be explained by the need to strengthen the defense reaction when the probability of occurrence of the pathogen is higher.

The first miRNA (miR472) that targets the NLR genes was discovered in Arabidopsis [87]. Since then, many other miRNAs involved in the regulation of NLR genes for the control of resistance to both virus and fungus diseases have been described. In tobacco (*N. benthamiana*), the TIR-type *N* gene, which gives resistance to tobacco mosaic virus (TMV), is regulated by the miRNA cluster miR6019/6020. In particular, the 22-nt miR6019 cleaves the *N* transcripts in correspondence with the TIR coding region, which leads to the production of phasiRNAs in an *RDR6/DCL4*-dependent manner. Additionally, in this case, the resistance response strengthens as plants mature [88]. Another example is barley miR9863, which mediates the cleavage of *Mla1* NLR transcripts and induces phasiRNAs that repress immunity against powdery mildew fungus [89]. During powdery mildew infection, the fungus triggers a transient increase in *Mla1* transcript levels, followed by sustained induction of miR9863 and of the corresponding phasiRNAs during the late phase of infection, when *Mla1* mRNA accumulation is restored to nearly basal levels [86].

Families of miRNA exist that comprise different isoforms. An example is the miR482/miR2118 group, for which wide variability has been reported across species for the number of isoforms. While a single miRNA is present in the Arabidopsis genome [90], multiple isoforms have been annotated in tomato, which are argued to allow more effective silencing of the NLR transcripts when compared with Arabidopsis. In tomato, miR482/miR2118 are the predominant members that regulate *R* genes. Out of the NLR genes that have been annotated in the tomato genome, 58 are predicted to be miR482-targets, and most of these are non-TIR-type NLRs [90]. Tomato plants infected with turnip crinkle virus (TCV), cucumber mosaic virus (CMV), and tobacco rattle virus (TRV) all showed a suppressed miR482-mediated silencing cascade, which resulted in increased expression of the corresponding resistance genes [90]. Although some miR482 isoforms were predicted to target a unique NLR transcript, most miR482 members have multiple NLR targets, as they are specifically designed to interact with the region coding for the P-loop (also called Walker A), a conserved motif in many R proteins. In this way, a few miRNAs can regulate the large NLR-coding gene repertoire. Nevertheless, among the mature miR482 sequences, there are six isoforms that correspond to wobble or variable sequences in NLR transcripts, for more specific regulation. Likewise, excessive production of miR482e in potato plants compromises NLR protein activity, which results in plants that are hypersensitive to *Verticillium dahliae* infection [91]. In monocots like rice, maize, and *Brachypodium*, in contrast, miR482/miR2118 targets noncoding transcripts, instigating secondary phasiRNA production mainly in reproductive tissues [92,93,94,95]. This dramatic functional divergence of a miRNA is rare in plants, and is perhaps unique to this miRNA family.

As well as their role in responses to pathogens, NLR genes are also involved in plant interactions with nonpathogenic microorganisms. Legumes are a well-studied system, as unique plants that can establish symbiotic relationships with nitrogen-fixing bacteria (rhizobia) within nodules of their root system. An integrated analysis of small RNAs, target prediction, and degradome data has provided some insights into the role of miRNAs that target NLRs in beneficial microbial interactions. In *Medicago truncatula*, three 22-nt miRNA families (miR1507, miR2109, miR2118) target conserved domains in NLRs and trigger the production of phasiRNAs [96]. In soybean nodules, it has been demonstrated that miR482, miR1507, and miR1510 are master regulators of the NLR family, through the production of phased small RNAs [85]. Furthermore, miR482, miR1512, and miR1515 are induced during rhizobia infection, and their transgenic expression increases nodule numbers, which suggests that these miRNAs have roles in nodule development [96]. Indeed, to facilitate beneficial microbial interactions, the defense responses have to be limited in the presence of rhizobia, and the high expression of miRNAs might be responsible for the overall suppression of the NLR transcripts [85].

To date, miRNA-based regulation of NLR genes has been detected in dicot and monocot plants, in both annuals and perennials, which indicates the widespread and conserved mode of regulation of NLR transcripts [25]. This finding indicates the relevance of this mechanism in the control of NLR expression and homeostasis, and in a co-evolution process between NLR genes and the corresponding small RNAs, to balance the benefits and costs of the plant immune system.

## 6. Regulation at the Protein Level

NLR proteins are normally expressed at low levels and assume competent, inactive states in the absence of pathogen triggers. Their activation is essential for effective plant defenses based on hypersensitive responses, but fine control of their activity is necessary. There are examples of R proteins that are rapidly degraded at the onset of the hypersensitive response to limit the extent of cell death and the amplitude of the overall disease resistance response [97]. Analogously, as incompletely folded R proteins can trigger cell death, they enter a default ubiquitination and degradation pathway [98].

On the other hand, the over accumulation or constitutive activity of *R* gene products in the absence of the pathogen can result in autoimmune phenotypes, with spontaneous hypersensitive response leaf lesion formation, in combination with dwarf stature and anomalous development [99]. A known example is *snc1* (*suppressor of npr1-1, constitutive 1*), a gain-of-function mutant in a TIR-type NLR-type *R* gene that shows constitutive defense activation without pathogen attack, which results in autoimmune phenotypes that include dwarf stature, increased SNC1 protein stability (without any alterations in the corresponding gene expression), high defense marker gene expression, elevated accumulation of the defense hormone salicylic acid, and disease resistance [100]. The *ssi4* (*suppressor of salicylic acid insensitivity of npr1-5*, *4*) mutant showed analogous phenotypic traits, which again did not appear to be caused by overexpression of the *R* gene, but rather by a constitutively activated R protein as a consequence of the amino-acid substitution in the NLR [101].

The phenotypes of constitutively activated defenses recurrently arise by disruption of negative control systems that act at several different stages of the immune response, and have been revealed by analysis of these autoimmune mutants. However, the molecular mechanisms that regulate the abundance and activity of receptor proteins are far from being well-understood. Protein folding, compartmentalization, and nucleocytoplasmic trafficking play main roles in the control of NLR activity. R proteins need to be maintained in a folded, metastable inactive state until triggered by a stimulus. Moreover, as R proteins are highly variable and rapidly evolving, the control systems co-evolve with them to ensure specificity. During the evolutionary process, R proteins accumulate cryptic mutations that lead to unstable and/or inactive proteins if they are not hidden by a buffering system. However, based on evidence accumulated so far, only a few molecular components appear to be involved in the challenging issue of controlling R protein activity. These are related to the chaperone machinery and to the ubiquitin-26S proteasome system [102,103], two interconnected systems that are recurrently exploited by plants for fine and prompt regulation of protein activity through control of the folding and abundance of the target protein. For instance, NLR proteins were listed among ubiquitinated proteins in a large proteomic study, and ubiquitination sites were identified on some R proteins [104]. On the other hand, the correct functioning of NLR proteins is dependent on heat shock protein 90 (HSP90) [105] and the two co-chaperones, SGT1 (suppressor of G2 allele of skp1) [106,107] and RAR1 (required for Mla12 resistance 1) [108], in the complex called HRS (HSP90-RAR1-SGT1). A knock-out mutation or silencing of either one of the complex components typically results in severe depletion of the NLR proteins and the corresponding effector-triggered immunity defense responses. This complex functions as a generic hub for multiple and distinct *R* genes. Indeed, specific inhibition of HSP90 compromised the resistance against various pathogens [109], and *rar1* mutations in Arabidopsis caused a loss of resistance conferred by RPS5/RPM1, RPP5, and RLM1/RLM2 against the bacterial pathogen *Pseudomonas syringae* [110], the oomycete *Hyaloperonospora arabidopsis* [111], and *Leptosphaeria maculans* [112], respectively. Impaired activity of HRS components has shown analogous effects on resistance conferred by other R genes in both monocots and dicots, such as *Prf* in tomato [113], *Mla* in barley [114], *Lr21* in wheat [115], and *Rx* in potato [116]. Interestingly, mammalian homologs of HRS components are also involved in immune responses [117]. The relevance of the HRS complex in *R*-gene-mediated disease resistance suggested that the HRS-based interactome network is a means to identify new resistance genes against the most widespread diseases of crops, as done for wheat and barley [118,119]. On the other hand, new evidence about the structure of the HRS complex that is also bound to the NLR domains has suggested an intricate network of interactions between the different proteins involved and their specific domains that regulate the folding and metastable state of NLR proteins [120,121]. The stoichiometry of the HRS complex also suggests the dimerization of NLR proteins following their activation by the cognate effectors that induce conformational changes [122].

Within the complex, SGT1 is the most intriguing component. Several studies have highlighted its role in the control of the stability, activity, and nucleocytoplasmic balance of R proteins (e.g., RPS4 by Zhang et al. [123]; MLA by Bieri et al. [114]; Bs2/AvrBs2 by Leister [124]; and N by Hoser et al. [125]). On the other hand, the loss-of-function mutant *sgt1* shows restoration of the reduced accumulation of RPS5 in *rar1* mutants, and the accumulation of SNC1, RPS2, and RPS4 (resistant to *P. syringae 2 and 4*) [126]. As SGT1 is a member of the SCF-type (SKP1, cullin, F-box protein) ubiquitin ligase complexes in yeast [127], and associates with components of the SCF complex in plants [128], the phenotypes appear to be related to the regulatory functions of SGT1 on some ubiquitin–proteasome pathways. Although many E3 ubiquitin ligases have been reported in plant immune signaling [129], so far only one has been shown to target NLR proteins to degradation. This is CPR1 (constitutive expresser of PR genes 1), an F-box protein that interacts and regulates the turnover of NLR proteins through their degradation by the 26S proteasome system. Its mutation causes elevated protein levels of SNC1 and RPS2, and activation of defense responses, while it does not affect the stability of RPS4 [101,130]. As the full spectrum of R proteins that CPR1 targets is not known, how CPR1 executes its specificity on its R protein substrates is not clear. Overall, a dual function can be hypothesized for SGT1 that depends on the presence of the pathogen. It might act as an adaptor between NLR proteins and HSP90, to favor their correct folding, the maintenance of a recognition competent state, and the protection from degradation, likely promoting intramolecular interactions between the NB and LRR domains that maintain the inactive state [124]. Otherwise, SGT1 might regulate the maturation of NLR by promotion of its degradation by the ubiquitin-mediated proteolytic pathway. The tricky aspect of this model is that the most conserved portion of SGT1, the domain SGS, binds the highly variable LRR domain of NLR proteins, and thus further adaptor proteins might be necessary to ensure the specific recognition of target proteins. One possible candidate might be HSP70. For instance, cytoplasmic *Capsicum annuum* HSP70 (CaHSP70) significantly accumulates in pepper leaves and induces the hypersensitive response to *Xanthomonas campestris* pv. *vesicatoria* (*Xcv*) infection; vice versa, its silencing increases susceptibility to *Xcv* and alters the cell death response to *Xcv* infection [131]. Another candidate for the regulation of R protein activity in synergy with SGT1 is SRFR1 (suppressor of rps4-rld), a conserved TPR domain-containing protein that can physically interact with SGT1 [96], as well as with R proteins, including SNC1, RPS4, and RPS6 [132]. Indeed, elevated levels of SNC1, RPS2, and RPS4 were present in *srfr1* [96,133]. It can be hypothesized that SGT1 and SRFR1 serve as chaperones or scaffolds in the SCF^CPR1^ complex, through their protein–protein interaction interfaces, to guarantee the specificity of CPR1 toward a target group of R proteins.

More recently, further levels of regulation of NLRs based on other post-translational modifications have emerged as mechanisms for the generation or adjustment of important molecular signals in R-mediated resistance, also with potential connections to other plant responses. The *muse6* (*mutant, snc1-enhancing 6*) mutant revealed that alternative protein initiation and Nt-acetylation of SNC1 can alternatively serve as a degron for its ubiquitination, or as a signal for stabilization [134]. The loss-of-function *siz1* mutant also showed alterations in the SNC1 protein and autoimmune phenotype. SIZ1 encodes a small ubiquitin-like modifier (SUMO) E3 ligase, which regulates multiple plant processes, including immune responses [135]. *siz1* showed activation or over accumulation of SNC1, while *siz1* overexpression attenuated SNC1 protein levels [136]. SNC1 was shown to be sumoylated *in planta* and has four sumoylation sites and five putative SUMO-interaction motifs. In addition, SIZ1 was recently shown to act as a negative regulator of the SNC1-dependent immune response at high temperatures [137]. On the other hand, overexpression of the F-box protein CPR1 inhibits the growth defects and disease resistance of the *siz1* mutant [136]. As SIZ1 and SUMO1/2 both act as important positive regulators of growth, the functional relationships between the different post-translational modifications on SNC1 might be responsible for the trade-off between thermo-morphogenesis and immune responses [138].

## 7. Evolution of the NLR Gene Family

Plants and animals diverged from a common unicellular ancestor ~1.6–1.8 billion years ago [139,140]. Nevertheless, the similarity of the NLRs of metazoans and plant NLR resistance proteins (R-proteins) was noted soon after their discovery, which led to the hypothesis that they evolved from a common ancestor [141,142]. However, this hypothesis was recently rejected, with the suggestion that the domain architecture of NLRs evolved at least twice [143]. Plant R-proteins and metazoan NLRs share a common nucleotide binding site–leucine-rich repeat architecture. R-proteins can function directly or (more frequently) indirectly as receptors of pathogen-encoded effector proteins, which are often secreted by pathogens directly into host cells [1,144]. To overcome detection, such pathogen effectors evolve rapidly through gene loss/gain and mutation. To withstand the evolutionary battle, plants have evolved arrays of processes to maintain diversity of NLRs and to deploy efficient “pathogen traps”.

### 7.1. Evolutionary Mechanisms That Lead to NLR Diversification

The *NLR* gene family evolved through the conjunction of duplication, unequal crossing-over, ectopic recombination, and/or gene conversion events [145,146]. In addition, evidence of positive diversifying selection, an evolutionary force that favors the accumulation of mutations, is often found in NLRs. These processes contributed to make the NLR family one of the most variable gene families in plant genomes [146,147].

Evolutionary forces affect the variability of NLR gene families at different levels, which include copy number variation, presence/absence variation, and allelic variation. NLR copy numbers vary by orders of magnitude across species [29,148] (see Section 2), and these genes tend to be over represented in presence/absence variation regions [149,150]. This is an expected evolutionary response, as when a pathogen loses an effector recognized by an NLR, the fitness cost of the NLR can becomes unfavorable for the plant. The NLR presence is thus likely to be related to the frequency and severity of infection by a pathogen with the cognate effector, and to fluctuate with the frequency of effectors in pathogen populations [151]. Furthermore, for NLRs involved in indirect recognition, small changes to either NLR or its guardee can trigger autoimmunity. Such interactions would favor presence/absence variations, which have indeed been associated with lower accumulation of polymorphism [152].

Extreme allelic diversity has been observed for several NLR genes, including barley powdery mildew resistance *Mla* [153], stem rust resistance in wheat *Sr33* [154], Arabidopsis *Resistance to Peronospora Parasitica 13* (*RPP13*) [155], and lettuce *Resistance Gene Candidate 2* (*RGC2*) genes [156]. Allele diversification is associated with functional diversification; for example, more than 30 barley *Mla* functional alleles confer different specificities against barley powdery mildew strains [157], a function that can also be achieved through recognition of unrelated fungal effectors by the different *Mla* alleles [158]. Similarly, *Sr33* alleles provide valuable, intermediate levels of resistance to diverse *Puccinia graminis* f. sp. *tritici* races [159], and *RPP13* alleles confer resistance to different *Hyaloperonospora arabidopsidis* strains, a specificity that can be associated to recognition of unrelated *H. arabidopsidis* effectors by the different *RPP13* alleles [160]. This allelic diversity arises from three processes: point mutations, recombination, and domain fusions of canonical and non-canonical (i.e., integrated domains, NLR-IDs, see below) domains. The fusion of canonical domains is most likely driven by intragenic and intergenic sequence exchanges that shuffle polymorphic sites between individual genes (e.g., [161]), while for non-canonical domains, DNA transposition and/or ectopic recombination have been indicated as the most likely mechanisms of formation of NLR-IDs [162]. Non-synonymous mutations are enriched in LRRs when compared with NB-ARC, which suggests positive selection on the LRR domain [157,163] and purifying selection on the NB-ARC domain [28]. Specificity to pathogen effectors driven by LRR polymorphisms has been validated by experimental approaches [164], hence new pathogen recognition capabilities can be generated by LRR mutations that subsequently show signatures of positive selection.

Inter-allelic and paralog recombinations have major roles in NLR diversification. The *Lr21* leaf rust resistance gene was introgressed in bread wheat from *Aegilops tauschii*, the donor of the D genome of hexaploid bread wheat [165], and sequence analyses revealed that the functional *Lr21* allele was a chimera of two nonfunctional *Lr21* haplotypes (H1, H2) [166]. The active *Lr21* resistance gene can be experimentally reconstituted by intramolecular recombination of the inactive H1 and H2 haplotypes, thus indicating that novel, active resistance genes might have originally evolved in wild species by recombination of existing, non-functional haplotypes [167]. It has indeed been reported that the main difference that distinguishes the hexaploid wheat *Lr1* and the *Ae. tauschii* ortholog from the susceptible allele of *Lr1* is a polymorphic segment of 605 bp that encodes LRRs [168]. This most likely indicates that the *Lr1* gene in hexaploid wheat evolved through recombination in the LRR region of the diploid *Ae. tauschii* and was later introduced into bread wheat by gene flow based on hybridization. Current evidence indeed suggests that illegitimate recombination between LRR regions leads to shared repeats in paralogous NLRs [169]. These repeats facilitate inter-allelic recombination, which typically results in gene conversion [156]. The pre-disposition of LRRs for forming mismatches during recombination, which result in structural variability, might explain their convergent integration into immune receptors of metazoans and plants [143].

### 7.2. Evolution of Resistance Specificities

It was observed that different members of the same NLR family can rapidly diversify and adapt to perceive effectors of distantly related fungal pathogens. The *Mla* gene family confers race specific resistance against barley powdery mildew strains, and it was observed that protein sequences conferring resistance to different pathogens blast to the same MLA family member in the barley genome [167]. These include *Sr33* and *Sr50*, introgressed from rye and *Aegilops tauschii*, respectively, which both provide resistance to stem rust disease in wheat [154,170], as well as *Triticum monococcum MLA1*, *TmMLA1*) [153,157]. As wheat, barley, and rye share their last common ancestor about nine million years ago [170], this finding indicates that these different pathogen specificities evolved after wheat and barley divergence.

Similar evidence has been provided for *NLR* genes grouped in clusters. Resistance genes for downy mildew (*RPP8*) and for turnip crinkle virus (*HRT*) in Arabidopsis are highly homologous and locate at the same genomic positions in different accessions [171]. In the Arabidopsis accession Ler-0, the NLR genes RPP8 and RPH8A (unknown function) are located a few Kb from each other. HRT (present in the Arabidopsis accession Di-17) was identified as a product of recombination, as its LRR showed high similarity to RPH8A and its CC-NB-ARC region was highly similar to RPP8. A new specificity was thus originated after recombination of two linked genes, followed by gene conversion. Similarly, *Gpa2* and *Rx1*, two highly similar NLR genes located in the same R gene cluster of potato, confer resistance to two completely different pathogens, the potato cyst nematode *Globodera pallida* and potato virus X (PVX), respectively [172]. This evolutional flexibility of NLR genes to switch resistance specificities between phylogenetically unrelated pathogens was also recently supported through domain-swap experiments between *Gpa2* and *Rx1* in the LRR region. These allowed for the conversion of extreme virus resistance in the leaves into mild nematode resistance in the roots, and vice versa [173]. It has been postulated that the rapid adaptation to different pathogens might be the result of indirect recognition events [167], or considering that effectors with little sequence homology can have very similar protein structures [174], they might be recognized by the same or related NLR immune receptors.

Several experimental reports have indicated that resistance specificities reside in the LRR domain of *NLR* genes. Three-dimensional modeling of *Pm3*, the protein product of a multi-allelic gene that confers race-specific powdery mildew resistance in bread wheat, identified the LRR repeats as bearing the highest variability among the susceptible and resistant consensus sequences [167,175]. Most polymorphic amino acids occurred in LRRs 19–24 in the core motif LxxLxLxxN/C, and also in the loop regions of LRRs 20, 22 and 22a, which connect the core motifs. In contrast, the polymorphism hotspot in resistant *Pm3* variants was mostly confined to the core motif in the LRRs 25–28, specifically at solvent-exposed residues. The modeling implied that new *Pm3* resistance alleles would have evolved as a consequence of mutations in different LRR regions, and that both LRRs 19–24 and 25–29 might be involved in specific binding of the avirulence gene product, or of a putative guardee/decoy [167,175].

It has been reported that orthologous genes in different species that evolved in parallel over a long time can confer resistance to the same or similar pathogens. The powdery mildew form specialized on rye cannot infect wheat. Nevertheless, the effectiveness of *Pm8* (a powdery mildew resistance gene of rye that is an ortholog of wheat *Pm3*) toward wheat powdery mildew suggests that the molecular activity recognized by *Pm8* is functionally conserved in the two specialized mildews. Therefore, a comparative molecular study of the *Pm3*/*Pm8* interaction with mildew might reveal evolutionarily conserved virulence factors that are essential for the pathogen and that could ultimately be used to develop more durable resistance [167].

A paradigm of evolutionary diversification that affects resistance gene effectiveness involves incompatible NLR gene interactions in the offspring of crosses between particular plant individuals, which triggers an autoimmune-like response designated as hybrid necrosis [176]. This occurs when two incompatible genotypes are crossed. It has recently been speculated that such a process represents the result of an incompatible interaction between NLR genes [177]. For cereal plants, a related effect that leads to resistance suppression was reported for wheat plants, where the rye *Pm8* resistance gene effectiveness was suppressed in the presence of some (but not all) *Pm3* alleles. The suppression was ascribed to protein interactions that resulted in protein dimers or multimers that were ineffective for induction of the immune response [178,179].

Another recent paradigm shift in NLR evolution and function was the identification of NLRs with exogenous domain fusions that mimic targets of pathogen effectors (integrated domains, NLR-IDs), and therefore act as effector traps [144,162,167,174]. Examples of NLR-IDs include *A. thaliana* RRS1-WRKY and *Oryza sativa* RGA5-HMA. Both of these NLR-IDs require an additional genetically linked NLR, such as RPS4 and RGA4, respectively, for activation of disease resistance [180,181,182]. The RGA4/RGA5 and RRS1/RPS4 pairs are found as neighboring genes in inverse orientation and they share a common promoter, which suggests their co-regulation. The products of paired NLRs form protein complexes that are essential for the suppression of NLR auto-activation, as well as the initiation of the signaling cascade. While NLR-IDs are responsible for initial effector perception, their NLR partner is required for downstream signaling [180,181,183]. Furthermore, a NLR-ID major integration clade that is ancestral in grasses and with members that are often found on syntenic chromosomes was recently highlighted; here, DNA transposition and/or ectopic recombination were indicated as the most likely mechanisms of the NLR-ID formation [184].

## 8. Co-Evolution of NLR and Mechanisms of Regulation

Regulation at the level of mRNA splicing and stability have both been proposed to have roles not only in the fine regulation of NLR activity in response to biotic stresses, but also in the promotion of the evolution of this very large gene family. Alternative splicing can be considered as one of the mechanisms as the basis of this evolution, as the production of diverse protein products from a single gene can participate in amplification of resistance-gene variation and complexity [59]. For NMD, an interesting hypothesis was advanced very recently by Raxwal et al. [75]. The production of new TNL genes is promoted by structural rearrangements, duplications, and other mutations in both the coding and promoter regions. In the former, protein function can be directly affected, while in the latter, changes in gene expression levels can be observed. In any case, the generation of such new genes can have positive effects in terms of acquisition of new resistance features, or negative effects in terms of autoimmunity. For this reason, they can be negatively selected and eliminated from the gene pool. On the other hand, because of the above-mentioned mutations, these genes might also acquire features of NMD substrates. Thus, NMD can suppress the phenotypic manifestation of a portion of newly formed NLRs, to strongly limit their potentially harmful impact on fitness. Then, when a temporary alleviation of NMD occurs following a pathogen attack, these new NLR variants can be expressed. In the end, if they do contribute to pathogen defense, they can be positively selected.

A tight association was also found between the diversity of NLRs and miRNAs in a recent study, in which an analysis of NLRs of 70 land plants was coupled with extensive small RNA data [25]. The miRNAs typically target highly duplicated NLRs, so they act as master regulators and allow for an ‘economic’ means of regulation of this large gene family. Moreover, in light of plant evolution, it was shown that duplicated NLRs from different gene families periodically resulted in new miRNAs, often in quite distinct plant lineages. Most of these newly emerged miRNAs target the same conserved, encoded protein motif of NLRs, which is consistent with a model of convergent evolution for these miRNAs. These newly emerged miRNAs might then develop new target specificities. However, duplication and subsequent divergence of a pre-existing miRNA precursor has also been observed, which indicates that the extent of the evolutionary relationship between miRNAs and NLRs is complex and dynamic. Interestingly, families of heterogeneous NLRs are rarely targeted by miRNAs in the Poaceae and Brassicaceae genomes, which essentially lack the widespread targeting of NLRs by miR482/2118. Therefore, they represent unusual plant families with respect to this kind of regulation. Thus, with the exception of these two families, a co-evolutionary model of plant NLRs and miRNAs can be hypothesized, for plants to balance the benefits and costs of NLR defense genes [25].

## 9. New Biotechnology Approaches and Perspectives for Breeding

The identification of genes for resistance to pathogens is of outstanding importance in understanding the genetic and molecular mechanisms of resistance, and also to obtain plants with a high degree of resistance in the field, through genomic-based advanced strategies in breeding. Indeed, once a resistance gene has been cloned, two main approaches can be undertaken. On the one hand, the precise position on the chromosome and the sequence of the gene allow for the design of perfect molecular markers that are suitable for marker-assisted backcrosses or marker-assisted selection programs, without the risk of recombination events between the gene and the marker [185]. On the other hand, knowledge of the gene sequence and its precise position on the chromosome allows for direct modification of the plant genome through the insertion of the resistant allele using genetic transformation, or via gene modification/editing approaches (referred to as new plant breeding techniques; [186]). All of these approaches offer new opportunities for increasing the efficiency of using genetic resources as sources of genetic variability in crop breeding (including landraces and wild relatives).

The transgenic approach has been used previously to obtain plants that carry resistance to diseases based on barley *Rpg1* (reaction to *Puccinia graminis 1*), rice *Pi9*, *S. bulbocastanum RB2*, and soybean *Rps1-k* genes. All of these genes code for NLR proteins [187]. Furthermore, the transgenic approach followed by cross- and marker-assisted selection has been used to accumulate more than two alleles for the same locus in transgenic plants. As an example, this approach has been used for the *Pm3* gene (a non-TIR-type NLR) for resistance to powdery mildew in wheat, for which 17 resistance-promoting alleles have been identified [188,189]. Although gene pyramiding has great potential for the improvement of durable resistance in crops, this approach requires a long time and has the risk of losing the association between the resistant alleles and the molecular markers used, depending on the distance between them.

Cisgenesis and intragenesis use the same transformation techniques as transgenesis, but they provide for transferring genes and regulatory sequences from crossable, sexually compatible species (same species, closely related species). These can replace inherent alleles with superior counterparts or introduce new genes into the cultivated gene pool. The improvement of disease resistance through cisgenic and intragenic approaches has been successfully carried out in apple, potato, strawberry, and grapevine, by either transfer of resistance genes from related wild species, or overexpression of those already present within the crop itself. Recently, true cisgenic apples resistant to scab were developed through transfer of the *HcrVf2* (Rvi6) gene from the wild apple *Malus floribunda* (including its own promoter and terminator) into apple cv. Gala [190,191]. There are also similar examples of genes that code NLRs, and for the coiled-coil subgroup in particular. Indeed, a cisgenic resistant apple line of the fire blight susceptible cultivar ‘Gala Galaxy’ was obtained using the cisgene *FB_MR5* from wild apple *Malus × robusta 5 (Mr5)*, controlled by its native regulatory sequences [192]. Moreover, two broad spectrum potato late blight *R* genes, *Rpi-sto1* and *Rpi-vnt1.1*, from the crossable wild species *Solanum stoloniferum* and *Solanum venturii*, respectively (including their native promoters and terminators), were successfully transferred into three commercial potato varieties without using marker genes. The resulting cisgenic potatoes (*Solanum tuberosum*) expressed a stack of late blight *R* genes, which conferred broad-spectrum resistance to late blight without affecting the characteristics of the original variety at maturity [193,194].

Cisgenic and intragenic approaches still have some drawbacks that limit their wider application. The random integration of cisgenes or intragenes into the host genome can potentially result in variability of gene expression because of negative position-dependent epigenetic regulation [191], or similarly to other transgenes, with interruption and silencing of local genes or other relevant sequences. Furthermore, a careful analysis of regulatory sequence types and sizes used in cisgenic and intragenic constructs is necessary. Finally, up to 80% of plants regenerated from cisgenic transformation experiments show integration of the vector backbone sequences [193]. Therefore, the use of minimal linear cassettes and biolistic gene delivery, instead of *Agrobacterium* transformation, might represent valid approaches to have “cleaner” transformations [195,196,197].

The most relevant and recent new plant breeding techniques are those that are dedicated to the promotion of site-specific mutagenesis, defined as whole genome editing. In such genome-editing approaches, different techniques can be used to delete, replace, or insert specific genomic sequences into a precise site, targeted through a sequence-specific guide RNA. These are all based on the induction of cuts in the double-stranded DNA in a preselected region of the genome, which is then ‘repaired’ with two different DNA repair processes: nonhomologous end-joining or homology-directed repair [198].

Some specific nucleases are used to induce the breaks in the double-stranded DNA, namely meganucleases, zinc finger nucleases, transcription activator-like effector nucleases (TALENs), and clustered regular interspaced short palindromic repeats/CRISPR-associated nucleases (CRISPR/Cas) [198,199,200]. The practical applicability of these genome-editing approaches depends on the availability of selected genotypes with good transformation/regeneration ability, to introduce relevant mutations into the primary gene pool, and subsequently to transfer these by conventional means [201,202,203]. Genome-editing approaches have significant applications to basic and applied research into crop plants [200,203].

Increasing pathogen resistance is a major area of application of genome editing in plant breeding. Susceptibility and resistant genes, genes that regulate the interactions between the effector and target, and genes that regulate the plant hormonal balance, can possibly be modified [204]. Bread wheat genotype resistant to powdery mildew disease was obtained by TALEN-mediated genome editing to inactivate the three homoeoalleles at the hexaploid mildew resistance locus *O* (*MLO*). The MLO protein is involved in negative regulation of vesicle-associated and actin-dependent defense pathways at the site of pathogen penetration [205]. Moreover, rice resistant to bacterial leaf blight was obtained using TALEN and CRISPR/Cas9-mediated knock-down of the sucrose transporter genes *OsSWEET13* and *OsSWEET14*. These genes encode members of the SWEET sucrose-efflux transporter family, which is involved in the efflux of sugar across the plasma membrane. *X. oryzae* pv. *oryzae* uses its endogenous TAL effectors, AvrXa7 or PthXo3, to activate the functional genes and thus divert sugars from the plant cell in favor of the pathogen [206,207]. CRISPR/Cas9-mediated target-gene mutation was also successfully used to modify the ethylene-dependent pathway that regulates the hormonal balance in rice. Deactivation of the ethylene-responsive factor (ERF) resulted in increased resistance to *Magnaporthe oryzae* [208,209]. Even if examples related to genes strictly belonging to the NLR gene family are still absent, the above-mentioned examples suggest that similar results can be achieved with these genes. It is important to underline that the genes described above have been made dysfunctional to obtain resistance. As loss of function of NLRs will not result in enhanced resistance; to obtain resistant alleles, their editing will require much more knowledge about the nucleotides to be modified and how to change them.

Genome-editing techniques preserve the native genomic structure, and therefore they are considered as safe technology for crop improvement. Despite this, their wider application and effectiveness to functional genomics and plant breeding presents some concerns related to the biosafety of crops obtained using these methods. One main concern is the possibility of off-target effects that are closely related either to the efficiency and specificity of the engineered nucleases, and especially to the choice of the target site. In any case, these concerns are limited when compared with the mutagenesis approach or classical gene transformation, and they can easily be surveyed through background selection and marker-assisted selection.

In conclusion, the use of both cisgene and intragene approaches and new genome-editing techniques can help to overcome concerns associated with the use of biotechnology in agriculture, and to provide new opportunities for more efficient use of genetic resources in crop breeding, through capitalizing on the information that the increasingly efficient genomics and phenomic platforms make available.

New plant breeding techniques allow for the precise modification of genes that underlie characters of interest, or their transfer into targeted varieties. In this way, the resulting genotypes do not contain any exogenous DNA sequences and are similar to those that can be obtained using conventional breeding techniques, such as mutagenesis [210,211,212,213]. Therefore, the usefulness and impact of new plant breeding techniques open innovative perspectives in plant breeding and might offer solutions for specific breeding aims. For this reason, they are under review worldwide to assess their possible exclusion from the current regulation systems for genetically modified plants [214], and the USDA has decided not to regulate CRISPR-edited crops (https://www.the-scientist.com/?articles.view/articleNo/52209/title/USDA-Will-Not-Regulate-CRISPR-Edited-Crops/).

## 10. Conclusions

The acquisition of new knowledge of the *NLR* genes is of outstanding importance to set-up breeding programs to improve crop resistance to the most devastating pathogens. Even if new breeding technologies, such as genome editing, are not yet widely used for the introduction of resistant alleles into crop genomes, these represent tools that can be exploited in the very near future for this purpose. The recognition specificity or activity of NLRs can be modified to improve resistance to plant pathogens (e.g., [215,216,217]), and similar mutations can be introduced via genome editing. In many cases, the plant resistance to pathogens that is driven by NLR genes is linked to nucleotide allelic diversity, and is not durable because of the evolution of novel virulence factors that can overcome the resistant alleles. Recent studies have described the molecular mechanisms that are devoted to the fine regulation of NLR expression and activity as having a pivotal role in resistance to biotic stresses. Deep understanding of these mechanisms might, therefore, open new perspectives for crop improvement in terms of resistance to biotic stresses. Indeed, the key regulatory factors can become breeding targets that can be modulated to obtain durable resistance.
